# Screening for eukaryotic motifs in *Legionella pneumophila* reveals Smh1 as bacterial deacetylase of host histones

**DOI:** 10.1080/21505594.2022.2149973

**Published:** 2022-11-25

**Authors:** Stefanie M. Herbel, Lambert Moyon, Marvin Christ, Eslam M. Elsayed, Brian E. Caffrey, Silke Malmsheimer, Iwan Grin, Kerstin Hoffmann, Kristin Surmann, Sascha Blankenburg, Anna Lena Jung, Christina E. Herkt, Marco Borsò, Beyza Bozdag, Axel Imhof, Anke Becker, Samuel Wagner, Gert Bange, Uwe Völker, Wilhelm Bertrams, Annalisa Marsico, Bernd Schmeck

**Affiliations:** a Institute for Lung Research, Universities of Giessen and Marburg Lung Center (UGMLC), German Center for Lung Research (DZL), Philipps-University Marburg, Marburg, Germany; b Computational Health Center, Helmholtz Zentrum München, Neuherberg, Germany; c Department of Chemistry, Philipps-University Marburg, Marburg, Germany; d Center for Synthetic Microbiology (SYNMIKRO), Philipps-University Marburg, Marburg, Germany; e Department of Biology, Philipps-Universität Marburg, Marburg, Germany; f Department of Microbiology and Immunology, Faculty of Pharmacy, Zagazig University, Zagazig, Egypt; g Computational Molecular Biology, Max Planck Institute for Molecular Genetics, Berlin, Germany; h Interfaculty Institute of Microbiology and Infection Medicine (IMIT), University of Tübingen, Tübingen, Germany; i Department of Functional Genomics, Interfaculty Institute for Genetics and Functional Genomics, University Medicine Greifswald, Greifswald, Germany; j Zentrallabor für Proteinanalytik, BioMedical Center, Faculty of Medicine, Ludwig-Maximilians-University of Munich, Planegg-Martinsried; k German Center for Infection Research (DZIF), Partner-site Tübingen, Tübingen, Germany; l Max-Planck Institute for Terrestrial Microbiology, Marburg, Germany; m Department of Medicine, Pulmonary and Critical Care Medicine, University Medical Center Giessen and Marburg, Philipps-University, Member of the German Center for Lung Research (DZL), Marburg, Germany; n Institute for Lung Health (ILH), Justus-Liebig-University, Giessen, Germany; o Member of the German Center for Infectious Disease Research (DZIF), Marburg, Germany

**Keywords:** Infection, macrophage, *Legionella pneumophila*, histone-deacetylase, Smh1

## Abstract

*Legionella pneumophila* (*L.p*.) is a bacterial pathogen which is a common causative agent of pneumonia. In humans, it infects alveolar macrophages and transfers hundreds of virulence factors that interfere with cellular signalling pathways and the transcriptomic landscape to sustain its own replication. By this interaction, it has acquired eukaryote-like protein motifs by gene transfer events that partake in the pathogenicity of *Legionella*. In a computational screening approach for eukaryotic motifs in the transcriptome of *Legionella*, we identified the *L.p*. strain Corby protein ABQ55614 as putative histone-deacetylase and named it “suppressing modifier of histones 1” (Smh1). During infection, Smh1 is translocated from the *Legionella* vacuole into the host cytosol. When expressed in human macrophage THP-1 cells, Smh1 was localized predominantly in the nucleus, leading to broad histone H3 and H4 deacetylation, blunted expression of a large number of genes (*e.g*. IL-1β and IL-8), and fostered intracellular bacterial replication. *L.p*. with a Smh1 knockdown grew normally in media but showed a slight growth defect inside the host cell. Furthermore, Smh1 showed a very potent histone deacetylation activity *in vitro*, *e.g*. at H3K14, that could be inhibited by targeted mutation of the putative catalytic center inferred by analogy with eukaryotic HDAC8, and with the deacetylase inhibitor trichostatin A. In summary, Smh1 displays functional homology with class I/II type HDACs. We identified Smh1 as a new *Legionella* virulence factor with a eukaryote-like histone-deacetylase activity that moderates host gene expression and might pave the way for further histone modifications.

IMPORTANCE

*Legionella pneumophila (L.p.)* is a prominent bacterial pathogen, which is a common causative agent of pneumonia. In order to survive inside the host cell, the human macrophage, it profoundly interacts with host cell processes to advance its own replication. In this study, we identify a bacterial factor, Smh1, with yet unknown function as a host histone deacetylase. The activity of this factor in the host cell leads to attenuated gene expression and increased intracellular bacterial replication.

## Introduction

*Legionella pneumophila* (*L.p*.) is a leading cause of pneumonia. In 2019, pneumonia ranked first as the most lethal contagious disease worldwide, being responsible for 2.6 million deaths [1]. The overall death rate of an *L.p*. infection is estimated to lie between 5% and 10% [2]. *L.p*. is a Gram-negative, rod-shaped, and aerobic bacterium of the class of gammaproteobacteria, motile by a single polar flagellum [3]. Infection of amoeba is far more common than infection of humans, which are a dead-end host for *Legionella*. Once the infection is established, it causes Pontiac fever or the usually more serious Legionellosis. *L.p*. enters the human lung *via* inhalation of carrier aerosols [4,5]. In the alveoli, *L.p*. infects alveolar macrophages, which they use as replication reservoir [6,7]. Replication inside the macrophages involves the establishment of a protective *Legionella* containing vacuole (LCV), which releases bacterial effector proteins into the host cell by means of a type IV secretion system (T4SS) [8]. These factors serve the hijacking of the host cell and the creation of a permissive cellular framework for *L.p*. replication [9]. Some bacterial factors target the translation of host transcript [10,11]. Due to the intracellular part of its life cycle, *L.p*. has acquired eukaryotic DNA motifs by gene transfer events and is able to secrete proteins into the host cell during infection. One of these factors, RomA, has been shown previously to act as a methyl-transferase in the host’s epigenome [12]. This host interference is a crucial aspect of *L.p*. infection.

To shed more light on the complex inter-species intracellular networks that arise in the course of infection, we screened *Legionella* factors for similarities with eukaryotic functional motifs. This approach identified ABQ55614, encoded by LPC_1677, as a probable histone-deacetylase. We confirmed the deacetylase activity by independent experiments and therefore propose the name Smh1 (suppressing modifier of histones 1) for ABQ55614. We could show that upon expression of this factor in eukaryotic host cells, the pro-inflammatory response of host cells upon *L.p*. infection was significantly diminished on the transcriptional level.

## Results

### Bioinformatic identification of Smh1 as a putative eukaryotic-like histone-deacetylase

**Figure 1.**
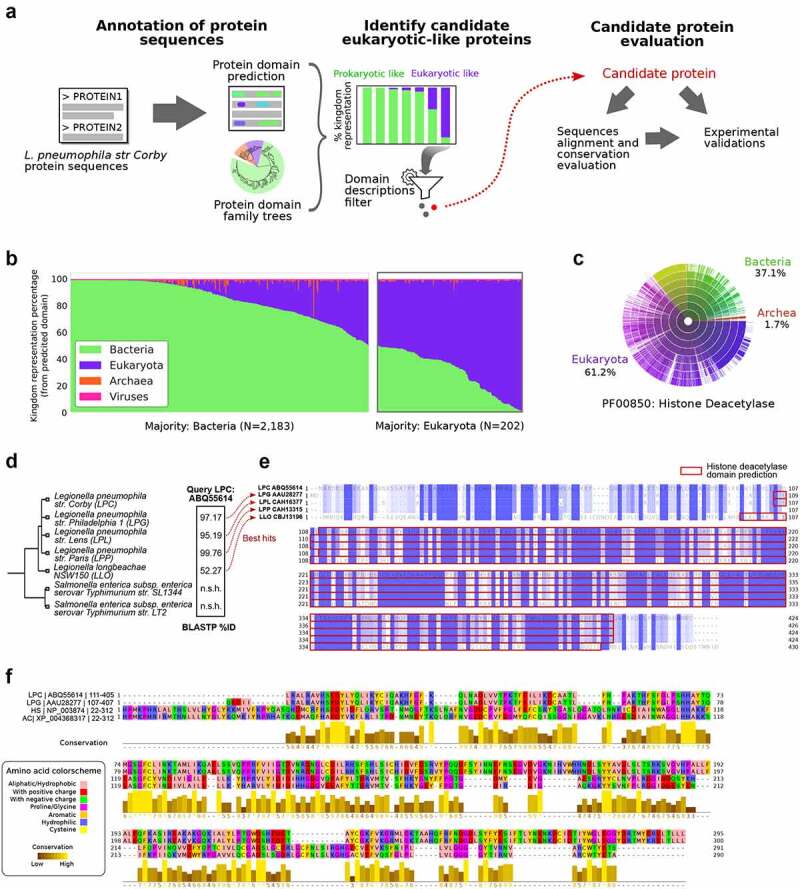
Screening of candidate eukaryotic-like proteins in *L.P*. strain Corby identifies Smh1 as a candidate histone-deacetylase. (a) Pipeline of analyses from the *in silico* annotation of protein sequences of *L.P*. strain Corby to the identification of candidate proteins from their eukaryotic-like protein domains, screened for enzymatic functions suggesting potential impact on the host regulatory machinery. (b) a total of 2,385 proteins were annotated with protein domains from Pfam database. Relative representation of annotated protein domain across the different kingdoms of life highlights a subset of 202 proteins bearing domains mostly represented in eukaryotic sequences. (c) Representation across kingdoms of the Pfam domain PF00850 “histone-deacetylase,” annotated in the sequence of the candidate protein ABQ55614 from *L.P*. strain Corby. (d) Results of the BLASTP search for homologous proteins to ABQ55614 in *L.P*. subspecies as well as *Salmonella* species. Percentage of sequence identity are reported for best hits; “n.S.h:” no significant hit. (e) Clustal omega multiple sequence alignment of the orthologs of ABQ55614 identified in *L.P*. subspecies. Blue colouring indicates conservation levels, while red frames indicate the independent predictions of the histone-deacetylase domains in each protein sequence. (f) Jalview visualization of the clustal omega alignment of histone-deacetylase domains extracted from *L.P*. proteins from strains *Corby* and *Philadelphia*, as well as from the Human Histone-deacetylase 3 (HDAC3, NCBI protein ID NP_003874) and the protein “XP_004368317” from *A. castellanii*. Amino acids are coloured to highlight physicochemical properties (Zappo colour scheme). Yellow barplots identify levels of conservation.

We applied a bioinformatic analyses pipeline (Figure 1a) aimed at predicting protein domains across all proteins of *L.p*. strain Corby, with the goal of better characterizing genes of unknown function, accounting for 40% of the *L.p*. Corby proteome. Predictions to identify candidate genes of interest were filtered for functional evaluations.

A total of 3,204 protein sequences from *L.p*. strain Corby were queried, and a total of 3,399 Pfam domains could be assigned to 2,385 proteins. We focused on domains bearing similarity to eukaryotic sequences, to extract a set of putative bacterial effector proteins that may hijack the host cell’s machinery through interaction with host protein partners, or act on the host’s regulatory programs. We identified 202 proteins with domains represented in most eukaryotic sequences (Figure 1b).

Filtering the protein list for over-representation of the assigned protein domain in eukaryotic sequences, as well as for domains related to enzymatic activities in transcriptional and post-transcriptional regulation, yielded a reduced set of 21 proteins, annotated with 15 unique protein domains.

Among these, we found the proteins with NCBI protein IDs ABQ57156, ABQ55118, and ABQ55614. The first protein was annotated with a “Histone methylation protein DOT1” protein domain (Pfam ID: PF08123) [13,14], represented by 90.3% of eukaryotic sequences. Although this domain is associated with histone 3 methylation of Lys79, likely involved in gene silencing at telomeres [15], it has recently been shown that inhibition of the host DOT1 L activity is associated with increased viral replication and decreased antiviral response [14]. The second protein (ABQ55118) was identified as bearing a SET domain-containing methyltransferase (95.9% of eukaryotic sequences). We identified the orthologue of this gene in *L.p*. strain Paris as the gene at locus lpp1683, which was previously characterized as the Dot/Icm type IV secreted effector RomA [16]. This factor was experimentally described as trimethylating K14 of histone H3 in the host nucleus and was identified as a required factor for bacterial replication in the host cell.

The third protein ABQ55614 was annotated with a histone-deacetylase domain (Pfam ID PF00850). This domain is found in most eukaryotic sequences (61.2%), while bacterial sequences represent 37.1% of all proteins bearing such domain, as reported in Pfam (Figure 1c). We decided to focus on the functional characterization of this protein, which is still unknown in the context of *L.p*. infection.

We first identified putative orthologues in other *L.p*. species and *Salmonella* species (Figure 1d), and their alignment highlighted high conservation of the amino-acid sequence (Figure 1e), especially in the histone-deacetylase domain regions, which were independently predicted in each of the orthologous sequences.

To further evaluate the putative function of this protein, we aligned the domain sequence of the protein ABQ55614 with the domain sequence from the orthologous sequence in *L.p*. strain Philadelphia, as well as domain sequences from two eukaryotic proteins: the human histone-deacetylase (HDAC) 3 (NCBI protein ID NP_003874) and one protein from *Acanthamoeba castellanii* strain Neff also annotated with this histone-deacetylase domain (NCBI protein ID: XP_004368317). The human HDAC3 protein was chosen as an example of a well characterized and experimentally validated histone-deacetylase. The XP_004368317 protein from *Amoeba*, found in one of the potential hosts of *L.p*. in its natural life cycle, might represent a protein whose function is mimicked by the candidate protein from *L.p*. strain Corby. The alignment of these sequences highlighted stretches of amino acids displaying high conservation, suggesting functional activity of this candidate histone deacetylase (Figure 1f).

This protein ABQ55614 was thus selected as a candidate for experimental validation. We suggest assigning the gene name “*smh1*” (suppressing modifier of histones 1) to this factor, which will be used in the present study. We detected Smh1 in THP-1 cells, which were infected with *L.p*., showing that it is expressed during the infection process (Figure S1).

### Smh1 is translocated from the LCV into the host cytosol in a T4SS dependent manner

**Figure 2.**
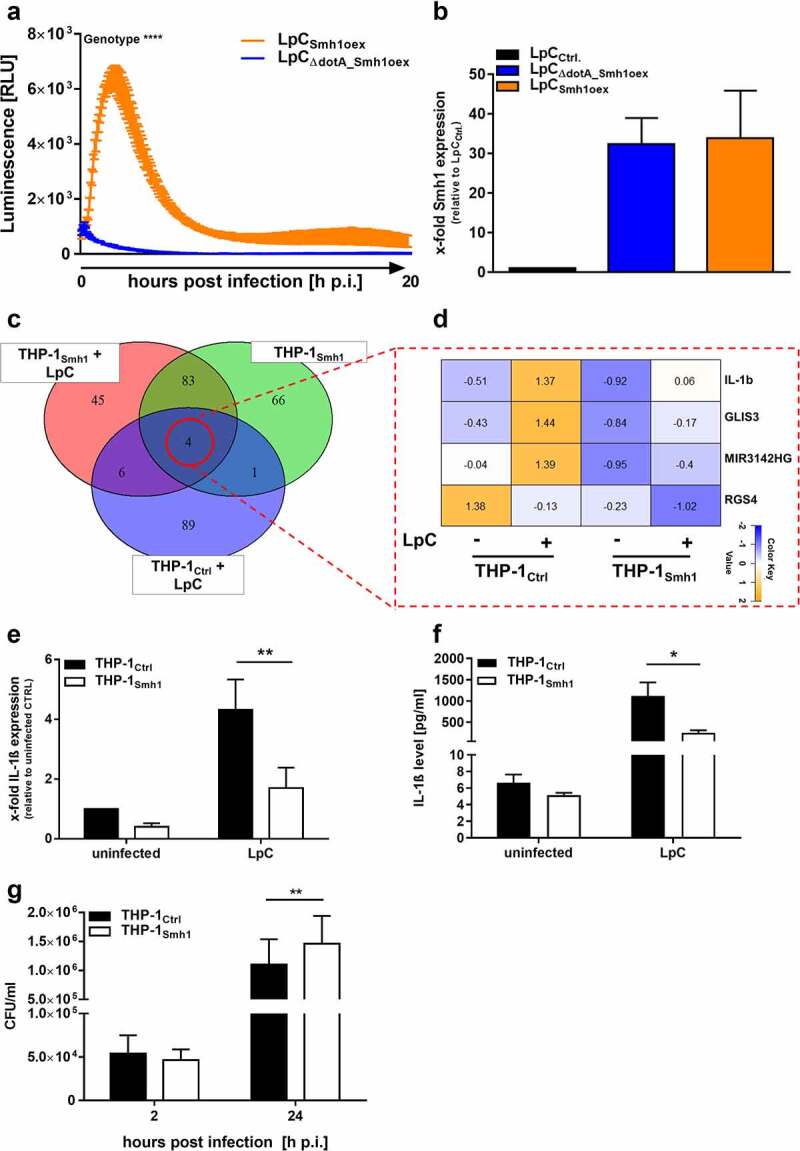
Effector protein Smh1 translocates into host cytosol and influences gene expression and *Legionella pneumophila* replication. (a) RAW 264.7 cells expressing cytosolic LgBit were infected with *L.P*. (T4SS-capable as well as the T4SS-incapable ΔdotA mutant) expressing HiBit-tagged Smh1 at MOI 50. Luminescence was detected over a period of 20 h. Two-way ANOVA with Sidak´s correction was performed and data are shown as mean ± SEM of at least three independent biological replicates. ****p ≤ 0.0001 for comparison between background genotypes. (b) Overexpression of Smh1 in LpC was verified by qPCR. (C-F) THP-1 cells expressing Smh1 (THP-1_Smh1_) and corresponding control cells (THP-1_Ctrl_) were stimulated with PMA (80 nM) for 72 h and infected with *L. pneumophila* Corby (LpC) at MOI 1 for 3 h (C-E) or 24 h (F) or left uninfected. (c) Venn representation shows significantly (p_adj_ <0.05) regulated genes after expression of Smh1 alone or additional LpC infection in comparison to infected Ctrl. Expression data for every section is provided in Table S1. (d) the four genes from the central Venn overlap are shown with their z-scores computed on normalized read counts from DeSeq2. (e) IL-1β expression was examined by qPCR and is shown as fold change of the uninfected Ctrl. (f) IL1β release was measured by ELISA. (g) PMA-differentiated THP-1 cells were infected with LpC at MOI 10. Bacterial replication was analysed by colony forming unit (CFU) Assay 2 and 24 hours post infection (h p.I.). (E-G) Two-way ANOVA with Sidak´s correction was performed and data are shown as mean + SEM of at least three independent biological replicates. *p ≤ 0.05; **p ≤ 0.01 (compared to infected Ctrl).

In order to establish the relevance of Smh1 as a modulator of the host response, we tested whether it is part of the array of factors that *L.p*. translocates from the LCV into the host cell. We used *L.p*. expressing a modified Smh1 molecule that carries one part of a luminescence reporter system. We could then show by split luciferase-based translocation assay that Smh1 indeed shuttles from the LCV into the host cytosol during infection with fully translocation-capable *L.p*., as evidenced by luminescence emission upon proximity of the cytosolic reporter component and tagged Smh1. In contrast, infection with a dotA *L.p*. mutant that does not have a functional T4SS does not lead to substantial occurrence of Smh1 in the cytosol (Figure 2a). Expression levels of tagged Smh1 were comparable between *L.p*. and dotA *L.p*. (Figure 2b).

### Expression of Smh1 reduced induction of pro-inflammatory gene expression and promoted Legionella replication

To assess the impact of Smh1 on eukaryotic host cells in the context of infection, THP1 cells stably expressing Smh1 (THP-1_Smh1_) (Figure S2) were infected with *L.p*. at MOI 1 for 3 h, and the whole transcriptomic landscape was explored by sequencing. We could establish that gene expression shifted substantially as a function of Smh1 abundance in contrast to THP-1_Ctrl_ cells (Figure 2(c)). Expression data for every diagram section is provided in Table S1. Of note, three of the four genes that were significantly regulated in all investigated conditions showed lessened induction upon concomitant infection and Smh1 expression (IL-1β, GLIS3, and MIR3142HG), while down-regulation of the fourth factor (RGS4) is accentuated by Smh1 expression (Figure 2(d)). We confirmed this observed pattern for IL-1β by qPCR and ELISA and saw a significant loss of induction on both transcriptomic and protein levels (Figures 2(e,f)). Furthermore, the expression of Smh1 led to reduced induction of IL-8, IL-6 and TNFα transcript upon infection, which was also observed to a weaker extent in uninfected cells (Figure S3A, C, D). The same infection procedure was carried out for 24 h, and secreted IL-8 was quantified by ELISA (Figure S3B). In line with the transcriptomic data, the amount of IL-8 was significantly reduced in the infected samples and subdued under baseline conditions when Smh1 was expressed. The effect of Smh1 on gene transcription also occurred in the context of a sterile TNFα stimulation, as IL-1β and IL-8 expression were significantly reduced after TNFα stimulation (Figures S3E, F). The impact of Smh1 expression in THP-1 cells on *Legionella* replication was investigated by counting colony forming units (CFUs) and monitored 2 h and 24 h post infection. Expression of Smh1 led to increased intracellular replication in comparison to control (Figure 2(g)).

### Smh1 acts as a histone deacetylase with impact on gene expression

**Figure 3.**
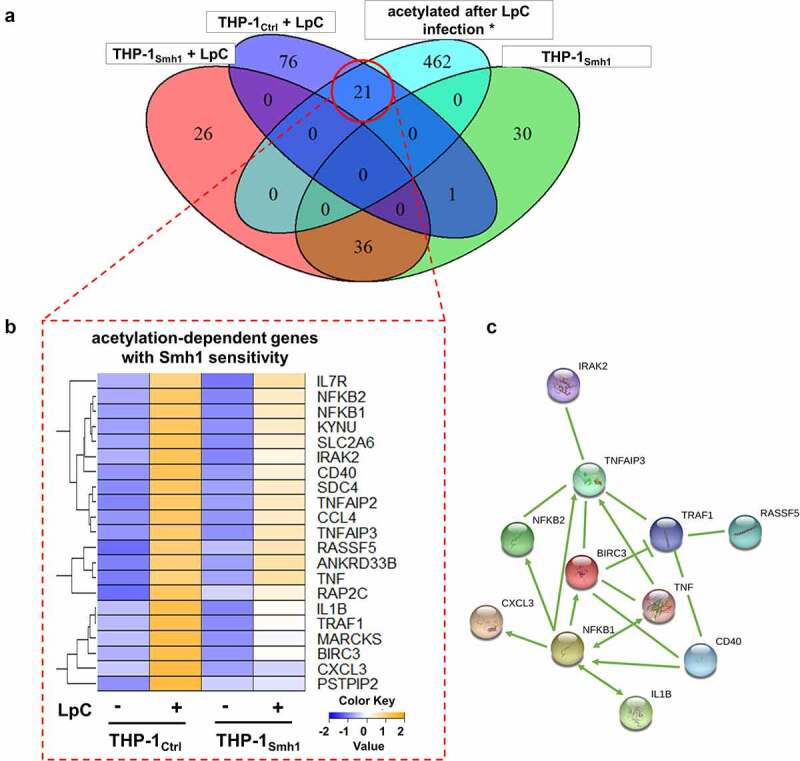
**Expression of Smh1 in THP-1 cells leads to loss of induction of key pro-inflammatory genes**. (**A**) Logic distribution of significantly (p_adj_ <0.05) upregulated genes in the indicated conditions in comparison to infected control (THP-1_Ctrl_ + LpC). Data on acetylated genes after LpC infection were taken from Du Bois *et al*. 2016 (*) [17]. Expression data for every section is provided in Table S2. (**B**) the 21 transcripts that fail to be regulated significantly in THP-1_Smh1_ cells are shown with their z-scores computed on normalized read counts from DeSeq2. (**C**) Ingenuity Pathway Analysis (IPA) revealed the interaction of 11 among the 21 proteins found to be acetylated after LpC infection which are also upregulated on transcript level in infected control cells but not in infected THP-1_Smh1_ cells. IPA filters were set to include only experimentally observed or high-confidence predicted interaction partners. Interaction types between nodes are provided in Table S3. Node aesthetics are from StringDB.

**Figure 4.**
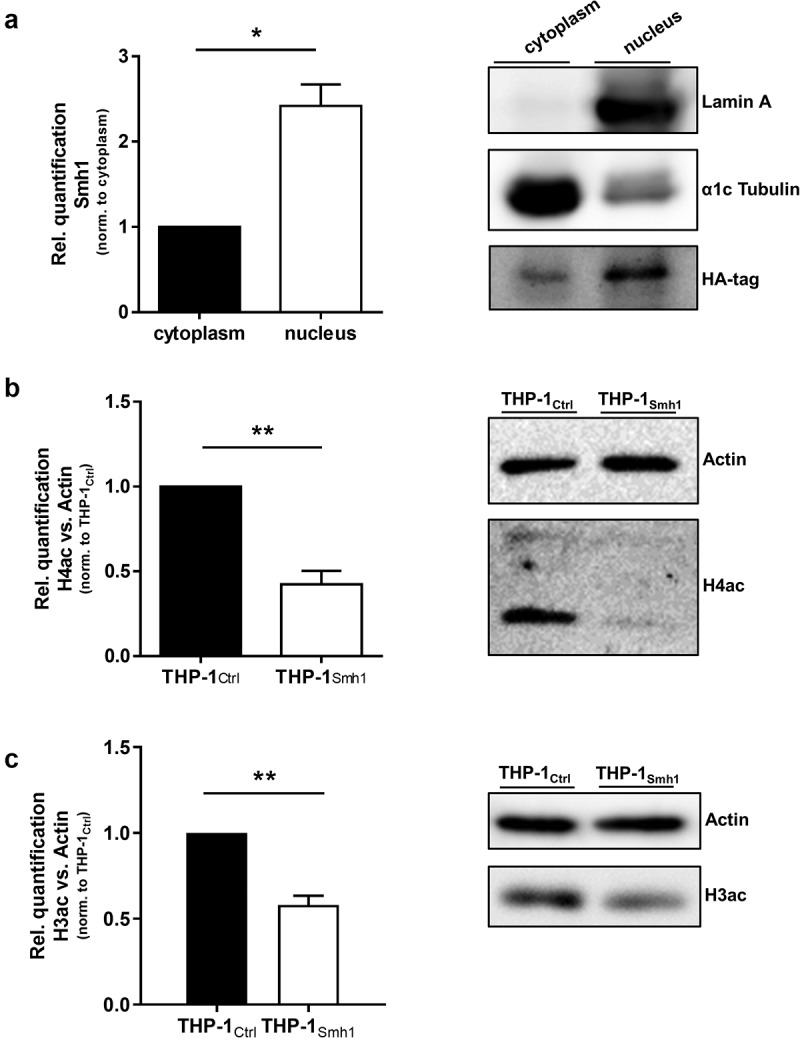
Smh1 is a histone-deacetylase and is active in the nucleus of the host cell where it targets histone 3 and 4. (a) THP-1_Smh1_ cells were stimulated with PMA (80 nM) for 72 h. Afterwards, the cytoplasmic and nucleic fractions were separated. Western Blot was performed against the Smh1-fused HA-tag. Lamin a was used as housekeeper for the nuclear fraction and α1c Tubulin for the cytosolic fraction. One representative blot of 3 is shown. The relative quantification of Smh1 is normalized to the cytosolic fraction. A paired t-test was performed. Data are shown as mean + SEM of three independent biological replicates. *p ≤ 0.05. THP-1_Smh1_ and corresponding control cells (THP-1_Ctrl_) were stimulated with PMA (80 nM) for 72 h. Function of Smh1 as a histone-deacetylase was confirmed by examination of histone 3 and 4 acetylation (H3ac and H4ac) by Western Blot (b and c). One representative blot of three independent biological replicates is shown. Relative quantification of H3ac and H4ac in comparison to actin is normalized to Ctrl. Unpaired t-test was performed, and data are shown as mean + SEM of three independent biological replicates. *p ≤ 0.05; **p ≤ 0.01.

We included a dataset on gene acetylation patterns upon *L.p*. infection, which we have published previously [17] into the present transcriptomic dataset to test whether gene expression and acetylation patterns can be traced to the same genes. We observed that the number of induced genes upon *L.p*. infection, which are also registered as acetylated, drops from 21 to zero as soon as Smh1 expression is included (Figure 3(a,b)). Expression data for every diagram section is provided in Table S2. Functional screening of these 21 candidates with Ingenuity Pathway Analysis (IPA) yielded a network with a highly enriched (p = 1.2 × 10^−8^) pro-inflammatory signature (Figure 3(c)). Interaction types between nodes are provided in Table S3. A prerequisite for the epigenetic activity of a protein (such as deacetylation) is its localization to the nucleus. Thus, we investigated the subcellular localization of a hemagglutinin (HA)-tagged variant of the Smh1 protein. Ensuring proper fractionation with markers for nucleus and cytoplasm (*i.e*., Lamin A and α1cTubulin, respectively), we found Smh1 predominantly in the nucleus of human THP-1 cells (Figure 4(a)). To experimentally confirm the hypothesized histone deacetylase activity of Smh1, we examined the overall histone-acetylation level of histone 4 and 3 in THP-1_Smh1_ cells by western blotting. After Smh1 expression, H4ac and H3ac showed a significant reduction of acetylation abundance by 50% (Figure 4(b,c)).

### Knockdown of smh1 selectively influences intracellular replication

**Figure 5.**
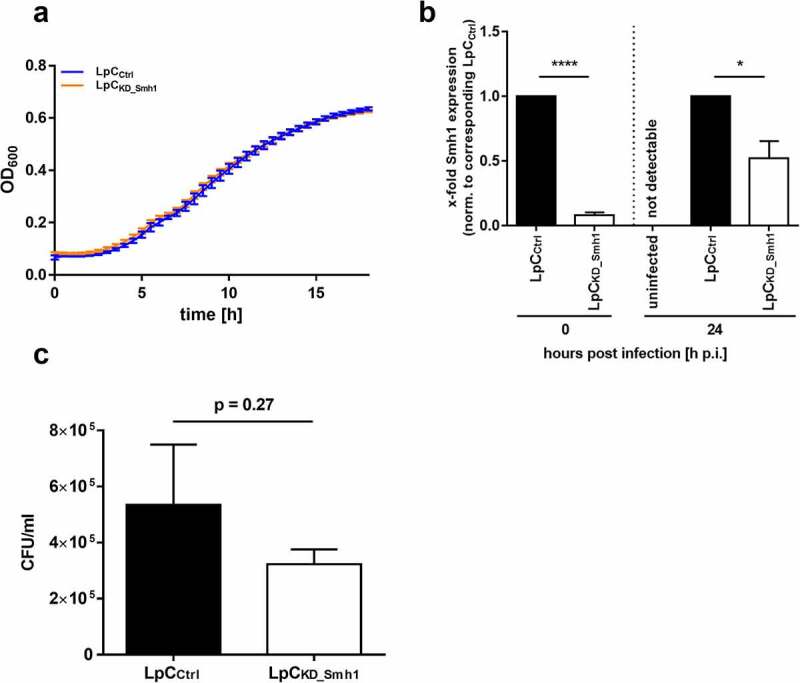
Knockdown of Smh1 in LpC only affects intracellular growth. (a) Growth of LpC with a CRISPRi – mediated Smh1 knockdown (LpC_kd_smh1_) was measured at a wavelength of 600 nm and a temperature of 37°C for 18 h. For comparison, LpC with a control vector (LpC_Ctrl_) were used. Data are shown as mean ± SEM of at least three independent biological replicates. (b + c) THP-1 cells were stimulated with PMA (80 nM) for 72 h. Afterwards, cells were infected with LpC_kd_smh1_ and LpC_Ctrl_ at MOI 1 for 24 h. (B) Smh1 knockdown was verified before from bacterial input material and 24 h post infection (h p.I.). One-way ANOVA was performed and data are shown as mean + SEM of at least three independent biological replicates. *p ≤ 0.05; ***p ≤ 0.001. (C) Bacterial replication was analysed by colony forming unit (CFU) assay 24 hours post infection (h p.I.). Paired t-test was performed and data are shown as mean + SEM of at least three independent biological replicates.

Next, we generated an anhydrotetracycline-induced smh1 knockdown in *L.p*. by CRISPRi. Growth of *L.p*. in liquid medium was not affected by the knockdown (Figure 5(a)). We then tested intracellular replication of the *L.p*. knockdown mutant. Knockdown was maintained through weakening over the whole experiment until 24 h (Figure 5(b)). We detected a slightly reduced intracellular growth of the knockdown mutant compared to control (Figure 5(c)). Albeit not significant (p = 0.27), this growth tendency is reciprocal to the observed growth advantage of *L.p*. in THP-1_Smh1_ cells. Cellular cytotoxicity of the used THP-1 cells is shown by LDH assay (Figure S4).

### Smh1 is a histone deacetylase analogous to class I or II HDACs, targeting H3K14 and H3K18

**Figure 6.**
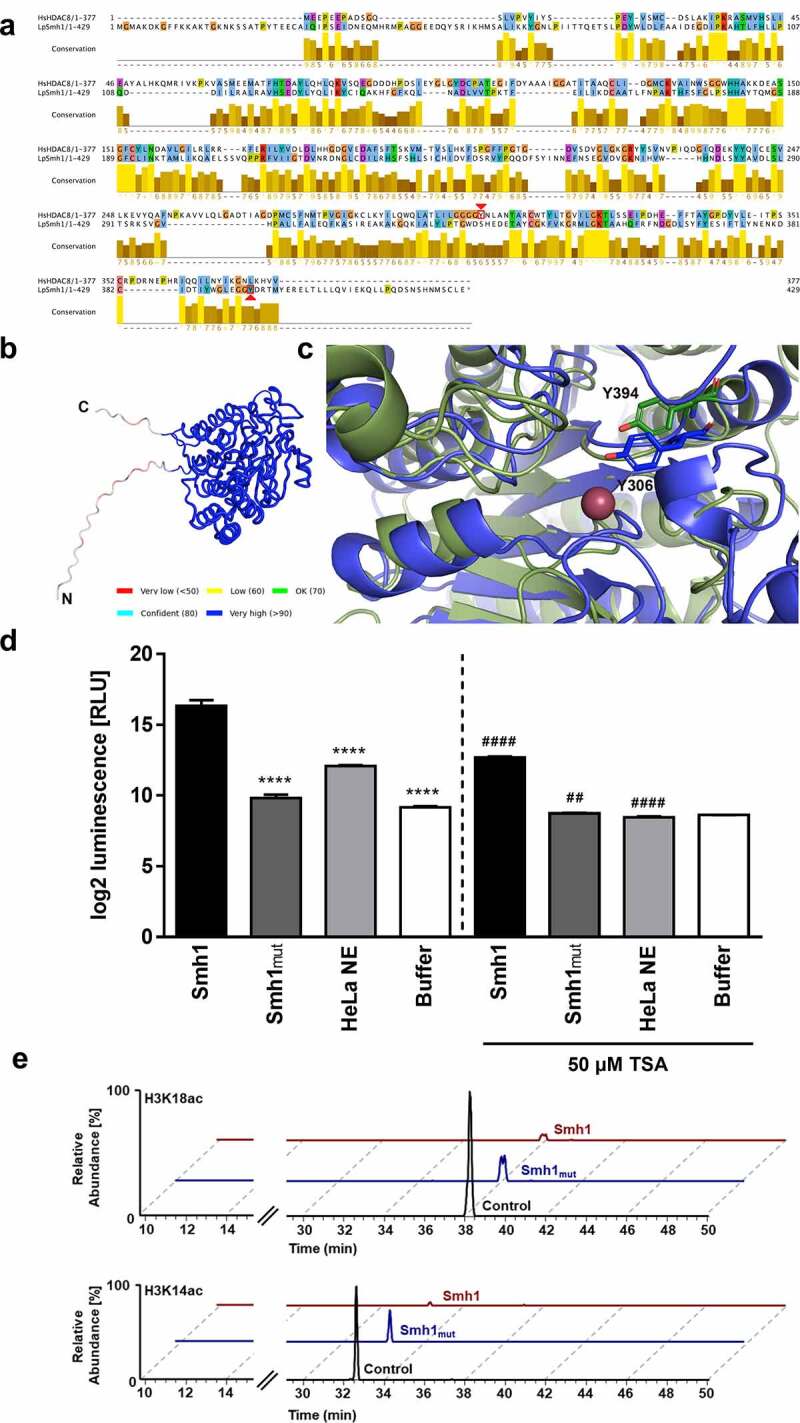
Targeted mutation of Smh1 leads to reduced HDAC function (a) the amino acid sequence alignment of HDAC8 (1^st^ row) and Smh1 (2^nd^ row) is shown. The bar chart under the sequences represents the conservation in both enzymes, 10 representing maximal conservation. The overall enzyme identity is 12.78%. The red arrows indicate the respective catalytic tyrosine in HDAC8 and Smh1. (b) Structure prediction of Smh1 with signal peptide. The colour represents the confidence of the model; the colour key is given under the structure model. Except for the *N*- and C-termini, the confidence-value is higher than 90% which represents a model with a high accuracy. (c) Superposition of the catalytic core of both enzymes. In blue is shown the enzyme form human and in green the one of *L. pneumophila* with the respective catalytic tyrosine. The reddish dot represents the zinc ion that is necessary for the catalytic mechanism. (d) a chemical HDAC activity assay specific for class I and II HDACs was performed with recombinant purified Smh1 as well as a Smh1 mutant (Smh1_mut_) expressed in *E. coli*. HeLa Nuclear Extract (NE) was used as positive control and buffer without Smh1 as negative control. For inhibition of Smh1, the enzyme was incubated for 30 minutes with 50 µm Trichostatin a (TSA). One-way ANOVA was performed, and data are shown as mean + SEM of three experiments. **** p ≤ 0.0001 (compared to Smh1 without TSA); ## p ≤ 0.01; #### p ≤ 0.0001 (compared to the respective condition without TSA). (e) Calf thymus histones were treated with recombinant purified Smh1 or a mutated version (Smh1_mut_). Subsequently mass spectrometry was performed. Relative abundances (percentages) were calculated for H3K18 and H3K14 acetylation (H3K18ac and H3K14ac). Untreated histones were used as control.

To confirm these data, we compared the putative structure of Smh1 to human HDACs and found, besides HDAC3, a conserved homology (12.78%) to eukaryotic histone deacetylase 8 (HDAC8; NP_001159890) (Figure 6(a)). HDAC8 is a class I histone deacetylase with high similarity to HDAC3 [18,19]. By using AlphaFold [20] we created a structural model of Smh1 with a confidence value of over 90% in the core of the protein, the low confidence areas being restricted to the flexible *N*- and C-termini (Figure 6(b)). Y306 is important for the catalytic activity of HDAC8. Moreover, by analyzing the sequences of HDAC8 and Smh1, the region around HDAC8-tyrosine 306 in Smh1 is not conserved (Figure 6(a)). Only through the structural superposition of HDAC8 and Smh1, the determination of Smh1-Y394 as catalytic tyrosine was possible (Figure 6(c), Smh1 in green, HDAC8 in blue). Thus, Smh1-Y394 was mutated to phenylalanine and a 40% reduction in activity was observed (Figure 6(d)), further strengthening our hypothesis that Smh1 is a histone deacetylase. By using a chemical HDAC activity assay detecting activity of class I and II HDACs, we demonstrated that recombinant purified Smh1 acts as a histone-deacetylase analogous to class I or II HDACs (Figure 6(d)). To support the function of Smh1 and especially the membership to class I or II HDACs, we inhibited Smh1 with Trichostatin A (TSA), a synthetic agent that specifically inhibits class I and II HDACs. TSA significantly reduced Smh1 HDAC activity in comparison to Smh1 alone (Figure 6(d)). Investigation of the Smh1 target spectrum by histone acetylation analysis revealed that Smh1 reduced H3K18 and H3K14 acetylation, among other sites, which was abrogated by the introduced mutation (Figure 6(e) and Figure S5). Thus, we conclude that Smh1 is a functional histone-deacetylase in human macrophages.

## Materials and methods

### Protein domain prediction

Protein sequences from *L.p*. strain Corby were extracted as a FASTA file downloaded from the NCBI database [21], using the GenBank assembly GCA_000092545.1. The InterProScan tool (version 5.50–84.0) [22] was applied with default parameters to process the FASTA file and produce an exhaustive prediction of protein domains over all sequences from *L.p*. strain Corby. We used the Pfam database (version 33.1) [23] as the resource for protein domains. Additional protein sequences (identified from downstream analyses of ortholog identification in related *L.p*. subspecies) were processed using the UniProtKB online database [24], notably for evaluating the presence of selected protein domains (specifically: the Pfam PF00850 “histone-deacetylase” domain, as identified in the selected candidate protein, see main results). This database was also used to query all proteins bearing this “histone-deacetylase” domain and to select eukaryotic proteins from organisms of interest (*Homo sapiens* and *A. castellanii*, see below).

### Putative eukaryotic domain identification and filtering

The assignment of protein domains to kingdoms (archaea, bacteria, eukaryota, and viruses) was done using predicted Pfam domains and using the species tree of domains from the Pfam database. For each protein domain in the database, we calculated the percentage of protein sequences harboring such a domain in each kingdom, using its associated species tree. Then, each of the *L.p*. strain Corby proteins was assigned to a representative kingdom by identifying the kingdom with the highest percentage value from their annotated protein domains. For protein sequences with multiple predicted domains, the predicted domain with the lowest prediction P-value (as reported by InterProScan for each domain predicted) was chosen for assigning a representative kingdom to the protein. We further filtered the list of proteins (and their predicted domains) by screening the protein domain descriptions, searching for the terms “transfer,” “histone,” “SET,” “deace,” “demet.” This aimed at identifying domains with putative enzymatic activities related to chromatin modulations of interest, indicating a potential role of the protein in transcriptional regulation of the host cell.

### Homologous genes identification and sequence alignments

We identified homologous proteins in related species using the BLASTP tool from the NCBI online webserver [25], with default parameters. For orthologue identification in *Legionella* species, we used BLASTP restricted to the proteomes of each of the following species: *L.p*. strain Philadelphia 1 (LPG), *L.p*. strain Lens (LPL), *L.p*. strain Paris (LPP), and *L. longbeachae* strain NSW150 (LLO). We also evaluated the presence of homologous sequences in species of *Salmonella enterica*: *Salmonella enterica subsp. enterica* serovar Typhimurium strain SL1344, and *S. enterica subsp. enterica* serovar Typhimurium strain LT2. Putative orthologues identified from the best hits in BLASTP were further confirmed through the OMA database of orthologues [26]. Orthologous sequences in *Legionella* species proteomes were then aligned using the Clustal Omega webservice from EMBL-EBI [27], with default parameter values except for the order of sequences, so that the *Legionella pneumophila* strain Corby sequence was taken as the reference. In addition to alignment of orthologous sequences, we proceeded to the alignment of sequences of distant organisms, looking for the presence of the protein domains of interest in their sequence. From querying the UniProtKB with the Pfam ID PF00850 (“histone-deacetylase” domain), we selected two eukaryotic protein sequences: *the* human Histone-deacetylase 3 (HDAC3, NCBI protein ID NP_003874), as well as a putative “histone-deacetylase 1” protein (NCBI protein ID: XP_004368317) from *A. castellanii* strain Neff. After extracting the protein sequence of the predicted domain from each sequence, we again proceeded to a multiple sequence alignment using the Clustal Omega webservice from the EMBL-EBI to evaluate the potential conservation of the enzymatic activity from the conservation in sequence.

### L. pneumophila culture

GFP-expressing *Legionella pneumophila* strain Corby was kindly provided by Prof. Dr K. Heuner, RKI, Berlin, Germany. For Smh1 knockdown and overexpression *Legionella pneumophila* Corby wildtype was used and kindly provided by the Robert Koch Institute, Berlin, Germany. In addition, for Smh1 overexpression ΔdotA *Legionella pneumophila* Corby was used kindly provided by Prof. Dr A. Flieger, Robert Koch Institute, Berlin, Germany. *L. pneumophila* was grown on buffered charcoal-yeast extract (BCYE) agar plates at 37°C and 5% CO_2_ for 3 d. For infection experiments, PMA-differentiated THP1 cells were infected with *L.p*. at indicated multiplicity of infection (MOI).

### Cell culture

THP1 cells (TIB-202™, ATCC®, Manassas, Virginia, USA) or THP-1 cells with a stable expression of Smh1 and the corresponding control cells were cultured with RPMI-1640 (Gibco^TM^, Life Technologies, Thermo Fisher Scientific, Carlsbad, USA) containing 10% (v/v) fetal calf serum (FCS, Gibco^TM^), 1% (w/v) glutamax, and 1% (w/v) sodium pyruvate (Gibco^TM^). Before infection, the cells were differentiated with 80 nM PMA (Sigma-Aldrich, St. Louis, Missouri, USA) for 72 h. HEK293T cells (CRL-3216™, ATCC®) as well as Raw 264.7 cells were cultured in DMEM (Gibco^TM^) containing 10% FCS. All the cells were cultured at 37°C, 5% CO_2_, and humidified atmosphere.

### Split Nanoluc-based translocation assay

Smh1 was cloned into pxDC61 using Gibson Assembly_®_ Cloning Kit (New England Biolabs, Ipswich, USA) according to the manufacturer’s protocol to tag it with the HA and HiBiT sequences. Details are given in the supplemental methods. Briefly, the coding sequence of Smh1 was generated from *L.p*. DNA by Phusion PCR using Phusion High-Fidelity DNA Polymerase (New England Biolabs) according to the manufacturer’s instructions (sense: 5´- CCTGATTATGCAGGATCCTCTGCCAAAGATAAAGGATTTTTTAAAAAAG-3´, antisense: 5´- CATCCGCCAAAACAGCCAAGCTTAACAGGACATATTATGCGAGTTG-3´). pxDC61 containing a HA-tag and a HiBiT sequence was amplified by Phusion High-Fidelity DNA Polymerase (New England Biolabs) according to the manufacturer’s instructions (sense: 5´- GCTTGGCTGTTTTGGCGGATGAGAGAAGATTTTC-3´, antisense: 5´- AGAGGATCCTGCATAATCAGGAACATCATACGGATATC-3´). The assembled plasmid was transformed into *E. coli* and verified. Afterwards, *L.p*. was electroporated with the pxDC61 plasmid containing Smh1 as described below. *L. pneumophila* strains carrying HiBiT-tagged Smh1 protein were grown for 2–3 d on BCYE-Agar plates supplemented with “BCYE Growth Supplements” and appropriate antibiotics. Gene expression was induced by incubation of bacteria o/n at 37°C on BCYE agar plates including 0.5 mM IPTG (Carl Roth). 4 × 10^6^ bacteria were diluted in HBSS + DrkBiT (1:1000) + 0.5 mM IPTG in a volume of 100 ml for infection of Raw 264.7 macrophages expressing LgBiT (kindly provided by Dr Erwin Bohn, Interfaculty Institute of Microbiology and Infection Medicine) at MOI 50. After centrifugation at 600 ×g for 10 min 25 µl of NanoGlo® live cell buffer (Promega, Madison, USA) supplemented with 1:20 of the extended live cell substrate (Promega) was added to each well. Luminescence signal was measured at 37°C every 5 min for around 20 h on a plate reader (Tecan Infinite M200 Pro).

### Transformation of Legionella pneumophila

*Legionella* was grown on BCYE agar plates at 37°C and 5% CO_2_ for 3 d. For transformation, 1 × 10^9^ Bacteria were resuspended in 1 ml ice-cold 10% glycerol and centrifuged for 5 min at 5.000 × g at 4°C. The supernatant was removed, and the pellet was washed twice in ice-cold 10% glycerol. The pellet was resuspended in 200 µl ice-cold 10% glycerol. Then, 100 µl bacteria plus the plasmid were transferred to a 2 mm gap electroporation cuvette (VWR, Darmstadt, Germany). Bacteria and plasmids were incubated for 30 min on ice and then electroporated at 3000 V, 200 ohm and 25 µF. *Legionella* were instantly incubated in 2 ml BYE broth liquid medium at 37°C on a shaking platform at 160 rpm for 3 hours. Bacteria were subsequently plated on BCYE agar plates with the appropriate selection antibiotics. Plates were incubated for 72 h at 37% and 5% CO_2_ for 3 d, and single cultures were retrieved.

### Cloning of smh1 into SparQ vector

The coding sequence of Smh1 was generated from *L.p*. cDNA by Phusion PCR using Phusion High-Fidelity DNA Polymerase (New England Biolabs) according to the manufacturer’s instructions. An HA-Tag was added by fusing the HA coding sequence to the reverse primer (sense: 5´-atcggaTTCGAAATGGCCAAAGATAAAGGATTTTTTAAAAAAG-3´, antisense: 5´-tccgatGCGGCCGCTTAAGCGTAATCTGGAACATCGTATGGGTAACAGGACATATTATGCGAGTTG-3´). The PCR fragments as well as the SparQ vector (Addgene, Watertown, USA) were digested with BstbI and NotI restriction enzymes (Thermo Fisher Scientific) and ligated with T4 DNA ligase (New England Biolabs) into the SparQ vector.

### Transfection of HEK293T cells and Lentivirus production

HEK293T (ATCC®) cells were transfected with the SparQ vector containing the sequence for Smh1 and a GFP sequence, the viral packaging vector psPAX2 and the envelope plasmid pVSV-G (Addgene) with Lipofectamine 2000 (Thermo Fisher Scientific)/Opti-MEM (gibco^TM^) mixture according to the manufacturer’s protocol. Lentivirus was produced, and virus-containing supernatant was collected every day for 72 h. Supernatant was filtered using a 45 µm microfilter and used for THP-1 cell transduction (see below).

### Transduction of THP-1 cells

THP-1 cells were transduced with the lentivirus from the filtered supernatant of the HEK293T cell culture (see above). Polybrene (4 µg/ml, Sigma-Aldrich) was added to improve the transduction efficacy. Cells were incubated for up to 6 d. GFP positive cells were isolated by flow cytometry. Smh1 expression was validated by qPCR (Figure S3) and by Western Blot against the HA-tag (Figure S4).

### Colony Forming Unit (CFU) Assay

To analyze bacterial replication, Smh1 expressing THP-1 cells and the corresponding control cells were infected with LpC as indicated in the respective figure legends. One-hour post infection (h p.i.), cells were washed and incubated with 50 µg/ml gentamycin for another 2 h. Upon 3-h post infection, the cells were incubated in fresh RPMI-1640 media. Samples were collected 2 and 24 h p.i.

Wildtype THP-1 cells were infected with Smh1 knockdown *L.p*. at MOI 1 for 24 h. Cells were lysed with 1% saponin (Carl Roth, Karlsruhe, Germany) and the diluted lysates were streaked on BCYE agar plates. Colonies were counted after 3 d at 37°C

### RNA extraction and qPCR

Total cellular RNA was isolated by phenol-chloroform extraction with TRI Reagent (Sigma-Aldrich). DNA digestion was performed with DNaseI (Roche, Mannheim, Germany) followed by RNA extraction with a phenol-chloroform-isoamyl alcohol mixture (Roti®-Aqua-P/C/I, Carl Roth). RNA was reverse-transcribed (High-Capacity cDNA Kit, Thermo Fisher Scientific, Carlsbad, USA), and quantitative real-time PCR was performed on a QuantStudio3 device (Thermo Fisher Scientific) with Luna_®_ universal qPCR master mix (New England Biolabs). Specific primers are described in Table 1. Smh1_oex primers were used to detect Smh1 expression in THP-1_Smh1_ cells. Smh1_Lp were used to detect Smh1 introduced by L.p. during infection of THP-1 cells.

**Table 1.** qPCR primers.

Target	Sense	Antisense
Smh1_oex	5´-CATCTATTGGGGCTTGGAAG-3´	5´-AGCGTAATCTGGAACATCGTATG-3´
Smh1_Lp	5´-AATGCCGATCTGGTCGTTAC-3´	5´-CGATTGACGTCGGTTCCTAT-3´
IL-8	5´-ACTGAGAGTGATTGAGAGTGGAC-3´	5´-AACCCTCTGCACCCAGTTTTC-3´
IL-1β	5´-ATGGAGCAACAAGTGGTGTTCTC-3´	5´-TCAACACGCAGGACAGGTACAG-3´
Rps18	5´-GCGGCGGAAAATAGCCTTTG-3´	5´-GATCACACGTTCCACCTCATC-3´
IL-6	5´-AATTCGGTACATCCTCGACGG-3´	5´-TTGGAAGGTTCAGGTTGTTTTCT-3´
TNFα	5´-GCTGCACTTTGGAGTGATCG-3´	5´-TCACTCGGGGTTCGAGAAGA-3´

### RNA sequencing and bioinformatics analysis

RNA was purified as described above. For library preparation and sequencing on an Illumina NextSeq 500 in the Philipps University Marburg Sequencing Core Facility, 500 ng RNA was used. Reads were mapped using the Qiagen CLC Workbench v. 10.0 and the human reference genome hg38. Differential gene expression was computed with the DeSeq2 Package v. 1.22.2 in the R v. 3.5.1. programming environment. Genes were considered differentially expressed at p_adj._ <0.05 (Benjamini-Hochberg corrected for multiple testing). For graphical representation of gene expression, transcripts per million (TPM) were computed and used for z score transformation. Biological interaction of chosen genes was investigated with Ingenuity Pathway Analysis (IPA). Data are available under the accession number GSE185936.

### ELISA

Commercial ELISA kits (OptEIA, BD Biosciences, Heidelberg, Germany) were used to detect IL-8 and IL-1β in cell supernatant according to the manufacturer’s instructions. The measurement was carried out on a Tecan Infinite M200 Pro (Thermo Fisher Scientific).

### Cellular fractionation

For separation of cytosol and nucleus protein fraction, differentiated THP-1 cells were washed and scraped into PBS (Capricorn Scientific GmbH, Ebsdorfergrund, Germany). After centrifugation (250 ×g, 4°C, 2 min) cells were lysed in Buffer 1 (10 mM Hepes pH 7.5, 10 mM KCL, 0.1 mM EDTA, 0,1 mM EGTA, 1× Protease inhibitor (Complete Mini Protease Inhibitor Cocktail, Roche, Germany), 0.5 mM DTT) and incubated at 4°C on ice (15 min). Lysed cells were drawn 7–8 times through a 26 G needle and centrifuged (4,600 ×g, 4°C, 2 min). Cytosolic supernatant was centrifuged (20,000 ×g, 20 min, 4°C) and was used for Western Blot. Nucleic pellets were washed two times with Buffer 1 and were lysed with Buffer 2 (20 mM Hepes pH 7.9, 400 mM NaCl, 1 mM EDTA, 1 mM EGTA, 1× Protease inhibitor (Roche), and 0.5 mM DTT) on a shaking incubator at 4°C for 1 h. Nucleic fractions were centrifuged (14,000 rpm, 4°C, 20 min) and supernatant was taken for Western Blot.

### Western Blot analysis

Total cellular proteins, cytoplasmic fraction, or nuclear fraction proteins were harvested by cell lysis, and protein concentration was measured by BCA assay (Thermo Fisher Scientific) according to manufacturer’s instructions. For protein separation, 10% or 15% SDS gels were used and 25–80 µg of protein per condition. Separated proteins were blotted on a nitrocellulose (Cytiva, Amersham, United Kingdom) or PVDF (Merck Millipore, Burlington, USA) membrane with the use of a wet blot or semi-dry system. The primary antibody (Table 2) was added at a 1:1,000 or 1:2,000 dilution and incubated overnight at 4°C on a tumbling shaker. The HRP-conjugated secondary antibody was added 1:2,000 for 1 h at room temperature on a tumbling shaker. After washing, protein signal was detected on the Bioluminescence and Chemiluminescence Imager (INTAS Science Imaging Instruments GmbH, Göttingen, Germany). Quantification of signal was performed by densitometric analysis, using the LabImage 1D software (Kapelan Bio-Imaging GmbH, Germany).

**Table 2.** Antibodies.

Antibody	Company	Article number
Anti-acetyl-Histone H4	Millipore (Burlington, Massachusetts, USA)	06-598
Anti-acetyl-Histone H3	Millipore (Burlington, Massachusetts, USA)	06-599
Anti-Histone H3	Abcam (Cambridge, England)	ab1791
Purified anti-HA. 11 Epitope Tag Antibody	BioLegend® (San Diego, Kalifornien, USA)	901533
α1c Tubulin	Santa Cruz Biotechnology (Dallas, Texas, USA)	sc-134239
Lamin A/C	Santa Cruz Biotechnology (Dallas, Texas, USA)	sc-20681
β-Actin	Santa Cruz Biotechnology (Dallas, Texas, USA)	sc-47778
Anti-mouse antibody: m-IgG_k_ BP-HRP	Santa Cruz Biotechnology (Dallas, Texas, USA)	sc -516102
Mouse anti-rabbit IgG	Cell Signaling Technology (Danvers, Massachusetts, USA)	5127S

### Cloning of smh1 into pEt24d

The coding sequence of *smh1* was generated from *L.p*. genome by polymerase chain reaction using Phusion High-Fidelity DNA polymerase (New England Biolabs) according to the manufacturer’s manual, with an elongation time of 1.5 min and a melting temperature of 58°C. The resulting PCR fragment was inserted into the BsaI restriction sites of pET24d plasmid, following the protocol of Weber et al. [28]. The resulting plasmid encodes *smh1* with a C-terminal hexahistidine-tag provided by the plasmid. For generating the *smh1*_*Y394F* mutation, overlap extension PCR was used, as described by Higuchi et al. [29] with the same settings as described before. (Sense: 5´ttaaggtctcccatgggcATGGCCAAAGATAAAGGATTTTTTAAAAAAGC3´, antisense: 5´ttaaggtctcctcgagACAGGACATATTATGCGAGTTGG3´ Primer Mutation: sense: 5’-GGAAGGTGGATTTGACAGGACCATGT-3‘, antisense: 5‘-CATGGTCCTGTCAAATCCACCTTCCA-3‘)

### Expression and purification of Smh1

For overexpression, the respective pET24d plasmid encoding the gene for *smh1* or *smh1_Y394F* was transformed *via* heat shock transformation after the protocol of New England Biolabs into chemically competent *E. coli* BL21 (DE3) (New England Biolabs). Cells were cultivated in lysogeny broth (LB) medium containing 50 µg/mL kanamycin, 100 µM ZnCl_2_ and 10 g/L D(+)-lactose-monohydrate for 20 h at 30°C and 180 rpm in a baffled flask before harvesting by centrifugation (3,500 g, 20 min, 4°C). The cell pellet was resuspended in lysis buffer (50 mM Tris-HCl pH 8.0, 3 mM MgCl2, 150 mM KCl, 5% glycerol, 0,25% NP-40, 1 mM 2-mercaptoethanol, protease inhibitor tablet (Roche Diagnostic GmbH)) and lysed with a microfluidizer (M110-L, Microfluidics) at 10,000 psi pressure. After centrifugation (47,850 ×g, 20 min, 4°C), the cleared lysate was loaded on a 1 mL HisTrap column (Cytiva) pre-equilibrated with 10 column volumes (CV) of lysis buffer. The column was washed with 10 CV of lysis buffer, and the protein was eluted with 10 CV of elution buffer (lysis buffer containing 100 mM imidazole, pH 8.0). The elution was concentrated with an Amicon Ultracel-10K (Millipore) to 500 µL and further purified via size-exclusion chromatography (SEC) on a HiLoad 26/600 Superdex 200 pg column (Cytiva) previously equilibrated with SEC buffer (50 mM TrisHCl pH 8.0, 150 mM KCl, 5% glycerol, 1 mM DTT). Fractions containing Smh1 were pooled and concentrated (Amicon Ultracel-10K (Millipore)), flash-frozen in liquid nitrogen, and stored until use at −80°C.

### HDAC-Glo I/II Assay

HDAC-GloTM I/II Assay (Promega, Madison, USA) was used as follows: 2.5 µg/ml of purified Smh1 and Smh1_mut_ were diluted in HDAC-Glo^TM^ I/II Buffer to a final dilution of 156.25 ng/ml. Then, 100 µl of the diluted enzyme were combined with 100 µl of HDAC-Glow^TM^ I/II Reagent according to manufacturer’s protocol, and the luminescence was measured using a Bioluminescence and Chemiluminescence Imager (Tecan Infinite M200 Pro). For inhibition of Smh1, 50 µM Trichostatin A (TSA) was used.

### Construction of the CRISPRi plasmids

For a targeted knockdown of Smh1, we generated CRISPRi plasmids [30] within the framework of the Marburg Collection, a recently published Golden Gate-based cloning toolbox [31]. A detailed description is provided in the supplementary methods. Briefly, the plasmid contained a catalytically inactive dCas9 under control of the inducible p_tet_ promotor, a gRNA expression cassette consisting of gRNA spacer and scaffold. The backbone carried a chloramphenicol resistance marker (CamR) and a RSF1010 origin of replication (supplementary methods Figures SM1 and SM2). Sequences are provided in the plasmid maps and the supplementary methods Tables SM1 and SM2. Assembly of the plasmids was performed in *E. coli* NEB Turbo. Construction of a target-specific plasmid was achieved by replacing a sfGFP dropout fragment with the gRNA spacer sequence by annealing two complementary oligonucleotides as shown in supplementary methods Table SM2. The oligonucleotides have been designed in the framework of CRISPRi browser (https://crispr-browser.pasteur.cloud/).

The sequence was confirmed by Sanger sequencing, and the plasmids were finally introduced into *Legionella pneumophila* by electroporation as described above. To induce the system, dCas9 expression was activated by anhydrotetracycline (aTC) at 100 ng/ml, which was added during *Legionella* growth on plates as well as during infection.

### Legionella pneumophila growth curve analysis

*Legionella* containing a CRISPRi-mediated Smh1 knockdown and control *Legionella* were grown on BCYE agar plates with 6 µg/ml Chloramphenicol and 100 ng/ml aTC at 37°C and 5% CO_2_ for 3 d. Afterwards, bacteria (OD_600_ 0.5) were resuspended in BYE broth liquid medium +6 µg/ml Chloramphenicol +100 ng/ml aTC at a final volume of 100 µl. Optical density was measured in a clear 96 well cell culture plate at 37°C every 30 min for 18 h by a plate reader (Tecan Infinite M200 Pro).

### Lactate dehydrogenase release (LDH) cytotoxicity assay

The supernatant of infected and uninfected THP-1 cells was diluted 1:10, and lactate dehydrogenase (LDH) release was measured with the Cytotoxicity Detection Kit (Roche) according to the manufacturer’s protocol on a plate reader (Tecan Infinite M200 Pro).

### Statistics

Data are shown as the mean + SEM of at least three independent experiments. Statistical significance was evaluated by two-way ANOVA with Sidak’s correction when more than two variables were analyzed. Unpaired or paired t-test was used for comparison of two different variables. p-values ≤0.05 were considered statistically significant.

## Discussion

In this study, we identified the *Legionella pneumophila* factor LPC_1677, which contains a histone-deacetylase domain and which we hence named *suppressing modifier of histones 1* (“Smh1”). We could show that Smh1 translocates from the LCV into the host cytosol in a T4SS-dependent manner. When ectopically expressed in THP-1 cells, Smh1 caused global deacetylation of Histone 3 and 4, along with a diminished pro-inflammatory cellular response upon *Legionella* infection. We were able to show that after Smh1 expression, more than 20 key pro-inflammatory genes were downregulated that had been previously established to be acetylated (hence activated) upon *L.p*. infection [17], suggesting that Smh1 deacetylated histones and consequently downregulated gene expression. We subsequently demonstrate sensitivity of intracellular *Legionella* replication to Smh1 levels. We could furthermore ascertain the HDAC activity of Smh1 in a chemical assay, where we could show that Smh1 reduced its activity when mutated or inhibited with TSA. Finally, we were able to demonstrate that Smh1 deacetylates, among others, at histone tail position H3K14.

The occurrence of eukaryote-like motifs such as Smh1 in *L.p*. argues for evolutionary conservation of genes that have been horizontally acquired in the course of infection. An advantage that the bacterium gains by a given factor exerts evolutionary pressure on keeping that factor. This advantage could *e.g.*, pertain interference with the cellular immune response. It has already been shown that a dysregulation of HDACs leads to a suppression of host genes, which play a crucial role in bacterial defense. For example, ankyrin A, an *Anaplasma phagocytophilium* effector protein, leads to an upregulation of HDAC1 in the host cell, which results in H3 deacetylation and suppression of defense genes [32,33]. Another example is the quorum-sensing molecule 2aminoacetophenone which is secreted by *Pseudomonas aeruginosa* and induces an HDAC1 upregulation in THP-1 cells. Afterwards, HDAC1 deacetylates H3K18. These are examples of bacterial effector proteins that indirectly deacetylate histones via HDACs from the host cell. In contrast to that, Smh1 seems to deacetylate global Histone 3 and 4 directly. It is already known that effector proteins from *L.p*. have a direct effect on histone modification in the host cell. Most prominently, the effector protein RomA trimethylates histone H3K14 [12]. In this publication, the authors speculate that acetylation and methylation of histone tail lysine residues happens in a competitive manner. We therefore hypothesized that the activity of Smh1 is necessary to clear the lysine residues of acetyl groups, to the effect that RomA can methylate them in order to efficiently shut down gene transcription for a more efficient intracellular bacterial replication. We investigated Smh1 target sites and could show that Smh1 indeed deacetylates H3K14. There is no specificity for that site, however, as we observed similar activity toward H4 and H3K18. The additional observation of a reduced Smh1 HDAC activity upon mutation of the catalytic site of Smh1, which we inferred from a previous publication about HDAC8 [18], indicates that Smh1 belongs to class I HDACs. Furthermore, the sensitivity of Smh1 to TSA argues for a zinc dependency of Smh1, as TSA exerts its inhibitory effect on HDACs by zinc ion sequestration [34].

The T4SS translocates over 330 effector proteins into the host cell. These factors interact with the host cell in many different ways to foster bacterial replication [35]. Despite this large array of factors, epigenetic regulation of host gene expression has only been shown in the case of RomA and hereby Smh1. While histone-deacetylase-like bacterial factors have already been described [36–38], we present the first case of such an enzyme in *Legionella*, which acts potentially in concert with RomA.

A limitation of our study is that we did not achieve a genetic knockout of *smh1* in *Legionella* with a methodology that we routinely use for knockout of other *L.p*. genes. The occurrence of strain-specific retroelements in the genome as shown for *Legionella* Corby [39] might influence the knockout efficiency at specific sites. However, the CRISPRi technique employed in this study led to a substantial knockdown at the time of infection, but this knockdown appeared to weaken after 24 h of infection. While we found the mode of action of Smh1, and show its translocation into the host cytosol, we think that we do not gauge the full impact this deletion has on Legionella replication, which we determined to be lessened at the 24 h time point. Improvement of the knockdown stability is hence necessary to explore the full consequence of a lack of Smh1 on the intracellular growth kinetics of *Legionella*. However, as the knockdown remained stable in liquid growth medium, where thus modified *Legionella* grew normally, we can rule out a function of Smh1 outside of the host cell.

In summary, we could establish that Smh1 is a determinant of intracellular *Legionella* replication via attenuation of host transcription, notably including IL-8 and IL-1β. We attribute this property to its HDAC function, which we show biologically and chemically. Our study now establishes the *Legionella* Smh1 protein as a deacetylase, which directly interferes with the chromatin modification machinery of the host cell, thus establishing a further case in the weaponry of pathogenic bacteria to manipulate their target host.

## Supplementary Material

Supplemental Material

## Data Availability

The data that support the findings of this study are openly available in NCBI Geo at https://www.ncbi.nlm.nih.gov/geo, reference number GSE185936.
