# Supplementary material for: Screening for eukaryotic motifs in Legionella pneumophila reveals Smh1 as bacterial deacetylase of host histones
Source: Virulence. 2022 Nov 25;13(1):2042–58. doi: 10.1080/21505594.2022.2149973 (PMC9704406; doi:10.1080/21505594.2022.2149973)
Supplement: Supplemental Material [file KVIR_A_2149973_SM9859.zip › supplementary/supplemental_methods.docx]

**Screening for eukaryotic motifs in *Legionella pneumophila* reveals Smh1 as bacterial deacetylase of host histones**

Stefanie M. Herbel, Lambert Moyon, Marvin Christ, Eslam M. Elsayed, Brian E. Caffrey, Silke Malmsheimer, Iwan Grin, Kerstin Hoffmann, Kristin Surmann, Sascha Blankenburg, Anna Lena Jung, Christina E. Herkt, Marco Borsò, Beyza Bozdag, Axel Imhof, Anke Becker, Samuel Wagner, Gert Bange, Uwe Völker, Wilhelm Bertrams, Annalisa Marsico, Bernd Schmeck

**Short title: Bacterial Smh1 deacetylates host histones**

## Construction of the CRISPRi plasmids

The basic plasmid was constructed with the modular Marburg cloning toolbox. It expresses the catalytically inactive dCas9 under control of the inducible p_tet_ promoter, a gRNA expression cassette consisting of gRNA spacer and scaffold where an adaptable gRNA spacer can replace a sfGFP dropout fragment. The backbone carries a chloramphenicol resistance marker (CamR) and a RSF1010 origin of replication (Figure SM1). Part sequences are provided in the plasmid maps and the Table SM1. Assembly of the plasmids was performed firstly in *E. coli* NEB Turbo*.* Adaptation for Smh1 target specificity (Figure SM2) was achieved by replacing a sfGFP dropout fragment with the gRNA spacer sequence by annealing two complementary oligonucleotides as shown in Table SM2. The oligonucleotides have been designed in the framework of the CRISPRi browser (<https://crispr-browser.pasteur.cloud/>).

The annealing reaction was set up by mixing 1.5 µL of each oligonucleotide (100 µM) with 5 µL T4-DNA ligase buffer (Thermo Scientific) in a total reaction volume of 50 µL. Reactions were incubated in a heat block at 95 °C for 15 min, before switching off the heat block for slowly cooling down the samples to room temperature (~1 h). Cloning reaction with the CRISPRi plasmid was set up with ~200 ng of plasmid, 3 µL annealed oligonucleotides, 0.5 µL of T4-DNA Ligase (5 Weiss U/µL, Thermo Scientific) and BsaI (10 U/µL) and 1 µL T4-DNA ligase buffer in a total reaction volume of 10 µL. Reactions were run in a thermocycler with 30 cycles of 37 °C (2 min) and 16 °C (5 min), followed by a final digestion step at 37 °C for 30 min and an enzyme denaturation step at 80 °C for 10 min. Transformation of *E. coli* NEB Turbo was performed with 5 µL of the cloning reactions by heat-shock transformation. The sequence was confirmed by Sanger sequencing and the plasmids were finally introduced into *Legionella pneumophila* by electroporation.

For inducing the system, *dcas9* expression was activated by adding anhydrotertracycline (aTC). Different concentrations of aTC have been tested and set to 100 ng/ml.


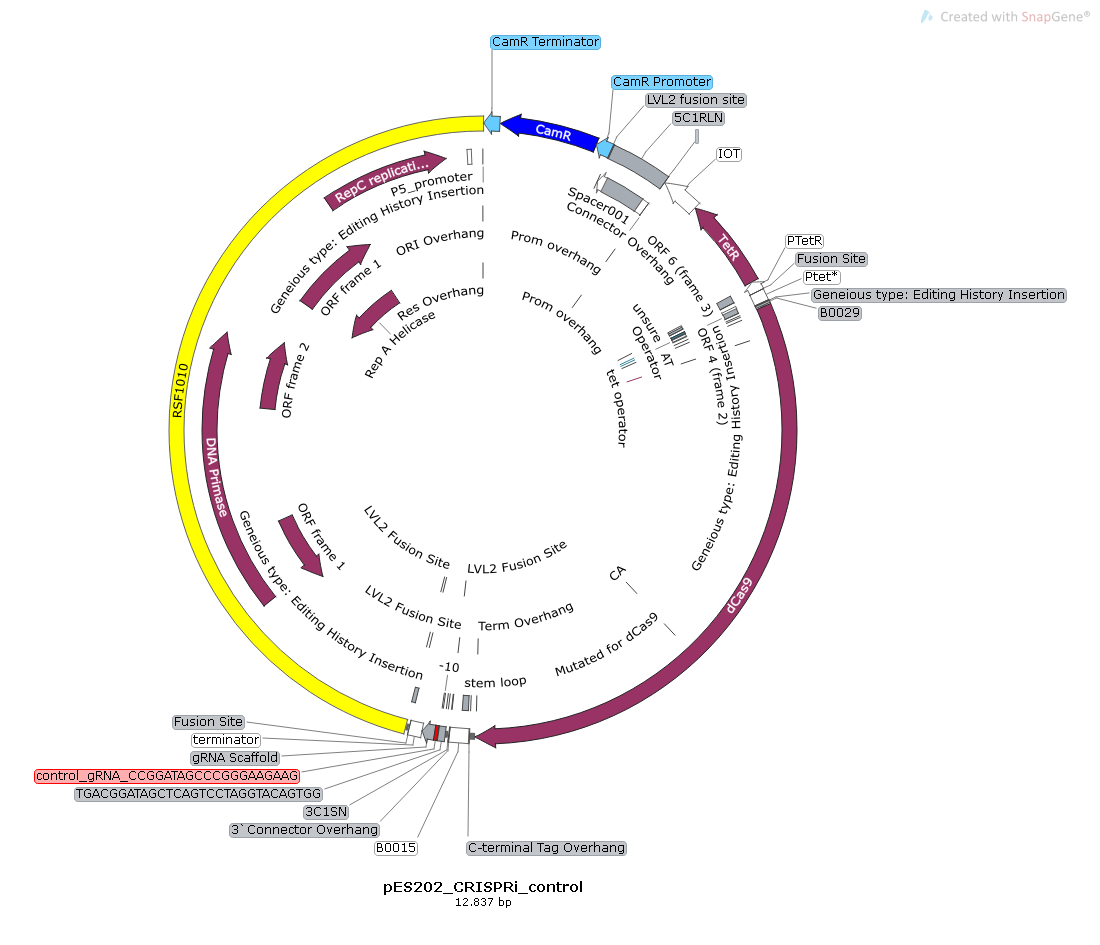


**Figure SM1:** CRISPRi control vector. Created with Snap Gene.


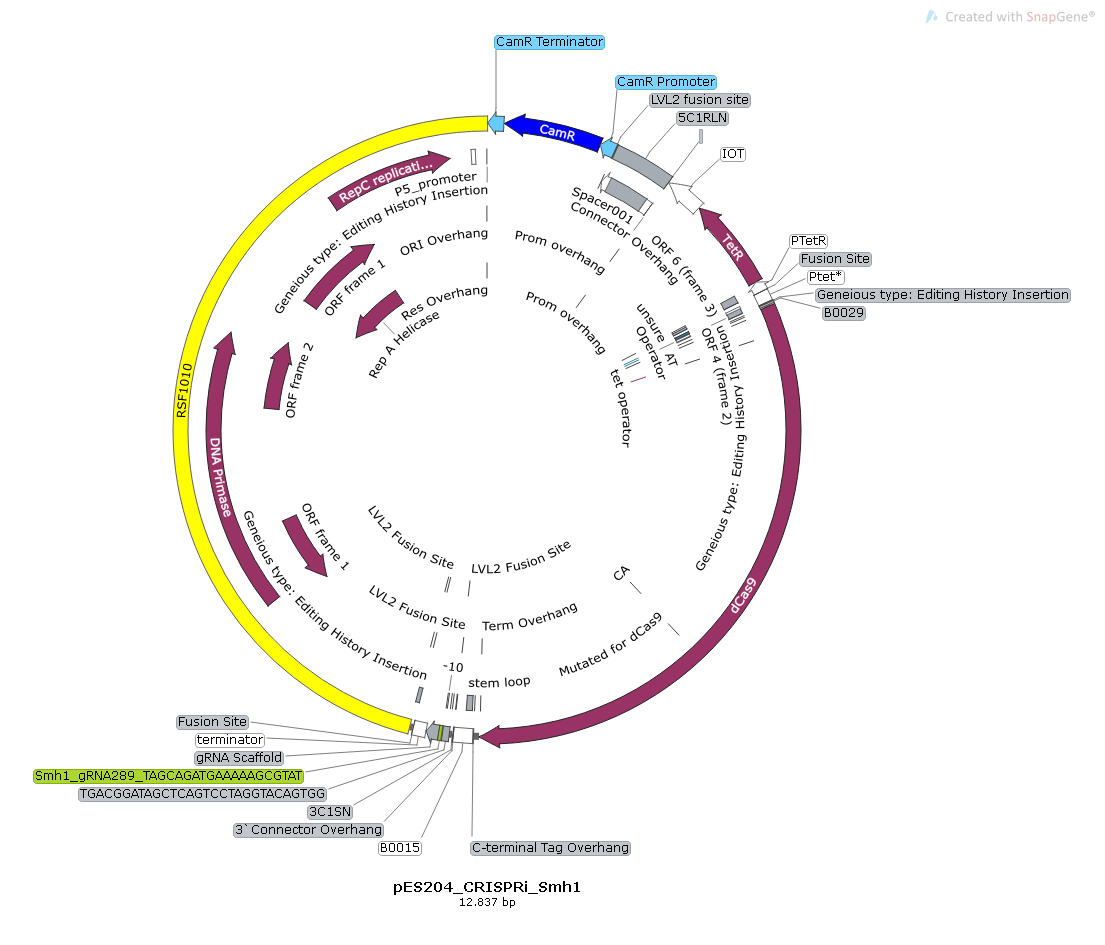


**Figure SM2:** Smh1 CRISPRi vector. Created with Snap Gene.

**Luminescence-based Translocation Assay:**

## Cloning of the *smh1* sequence into the pxDC61 vector

Smh1 was cloned into pxDC61 using Gibson Assembly^®^ Cloning Kit (New England Biolabs, Ipswich, USA) according to the manufacturer´s protocol. The coding sequence of Smh1 was generated from *L.p*. DNA by Phusion PCR using Phusion High-Fidelity DNA Polymerase (New England Biolabs) according to the manufacturer´s instructions (sense: 5´- CCTGATTATGCAGGATCCTCTGCCAAAGATAAAGGATTTTTTAAAAAAG-3´, antisense: 5´- CATCCGCCAAAACAGCCAAGCTTAACAGGACATATTATGCGAGTTG-3´). Amplification of pxDC61 containing a HA and HiBiT sequence was also done by Phusion High-Fidelity DNA Polymerase (New England Biolabs) according to the manufacturer´s instructions (sense: 5´- GCTTGGCTGTTTTGGCGGATGAGAGAAGATTTTC-3´, antisense: 5´- AGAGGATCCTGCATAATCAGGAACATCATACGGATATC-3´).

## Split Nanoluc-based translocation assay

One day prior to infection, Raw 264.7 macrophages expressing LgBiT in 100 µl DMEM + 10% FCS (Capricorn Scientific GmbH) were seeded in a 96 well cell culture plate (Greiner) at 8 x 10^4^ cells/well. *L. pneumophila* carrying HiBiT-tagged Smh1 protein was grown for 2-3 days on BCYE-Agar plates supplemented with “BCYE Growth Supplements” and appropriate antibiotics. Gene expression was induced by incubation of bacteria o/n at 37 °C on BCYE agar plates including 0.5 mM IPTG (Carl Roth). Bacteria (MOI 50) were diluted in HBSS + DrkBiT (1:1000) + 0.5 mM IPTG in a volume of 100 ml for infection of Raw 264.7 macrophages. After centrifugation at 800 x g for 10 minutes 25 µl of NanoGlo® live cell buffer (Promega, Madison, USA) supplemented with 1:20 of the extended live cell substrate (Promega) was added to each well. Luminescence signal was measured at 37 °C every 5 minutes for 20 hours by plate reader (Tecan Infinite M200 Pro).

## Histone acetylation analysis

Sample preparation for histone acetylation analysis were performed according to the EpiQMAx GmbH protocol (PMID 33790358). In short, calf thymus histones were treated for 2hr at 30°C with either wt or mutant enzyme, isolated and propionylated on beads using 200 mM N-(Propionyloxy) succinimide (Sigma) solution in ammonium bicarbonate for 30 min at 50℃ while shaking at 300 rpm. Following propionylation, proteins were digested with 50 ng of trypsin (Sigma) in 100mM ammonium bicarbonate overnight. Digested peptides were dried and re-suspended in 15 μl of 2% ACN, 0.3% TFA (PreOmics GmbH LC Loading buffer) and stored at -20°C until MS analysis. Before MS analysis samples were diluted 1:5 in LC loading buffer and a total of 5μl were injected into a nano-HPLC device (Thermo Fisher Scientific) using a gradient from 4% B to 90% B (solvent A 0.1% FA in water, solvent B 80% ACN, 0.1% FA in water) over 90 min at a flow rate of 300 nl/min in a C18 UHPCL column (Thermo Fisher Scientific). Data was acquired in top20 positive mode using a Q Exactive HF spectrometer (Thermo Fisher Scientific) to identify and quantify specific N-terminal peptides of histone H3 and histone H4 proteins and their PTMs. MS1 spectra were acquired in the m/z range 250-1600 with resolution 60,000 at m/z 400 (AGC target of 3x10^6^). MS2 spectra were acquired with resolution 15,000 to a target value of 2x10^5^, maximum IT 60ms, isolation 2 window 0.7 m/z and fragmented at 27% normalized collision energy. Typical mass spectrometric conditions were: spray voltage, 1.5kV; no sheath and auxiliary gas flow; heated capillary temperature, 250°C.

Raw files were searched with the Skyline software version 21.1  ([1](#_ENREF_1)) against histone H3 and H4 peptides and their respective PTMs with a precursor mass tolerance of 5 ppm. The chromatogram boundaries of +1, +2, +3 and +4 charged peaks were validated and the Total Area MS1 under the first 4 isotopomers was extracted and used for relative quantification and comparison between experimental groups. Relative abundances (percentages) were calculated as in the following example for H3K18 acetylation: %H3K18/23ac = (H3K18/K23ac)/ (H3K18/K23ac0 + H3K18/K23ac1 + H3K18/K23ac2) where "acN" indicates acetylation and "ac0" indicates unmodified. To illustrate changes upon Smh1 treatment the log2 fold change relative to untreated histones is shown.

**Bibliography:**

1. Pino LK*, et al.* (2020) The Skyline ecosystem: Informatics for quantitative mass spectrometry proteomics. *Mass spectrometry reviews* 39(3):229-244.
