# Supplementary figures and images for: Screening for eukaryotic motifs in Legionella pneumophila reveals Smh1 as bacterial deacetylase of host histones
Source: Virulence. 2022 Nov 25;13(1):2042–58. doi: 10.1080/21505594.2022.2149973 (PMC9704406; doi:10.1080/21505594.2022.2149973)

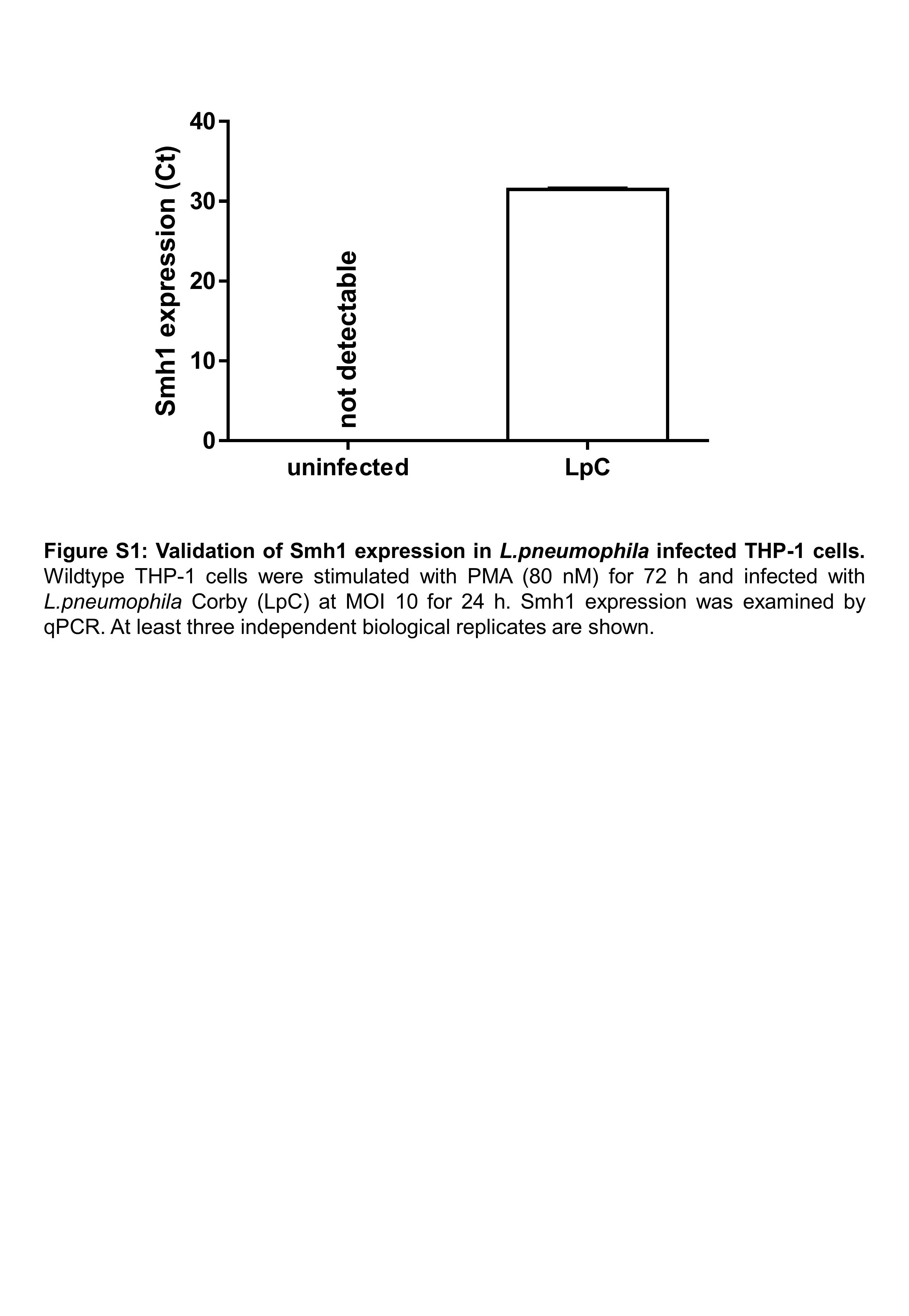

Supplement: Supplemental Material [file KVIR_A_2149973_SM9859.zip › supplementary/FigureS1.TIF]

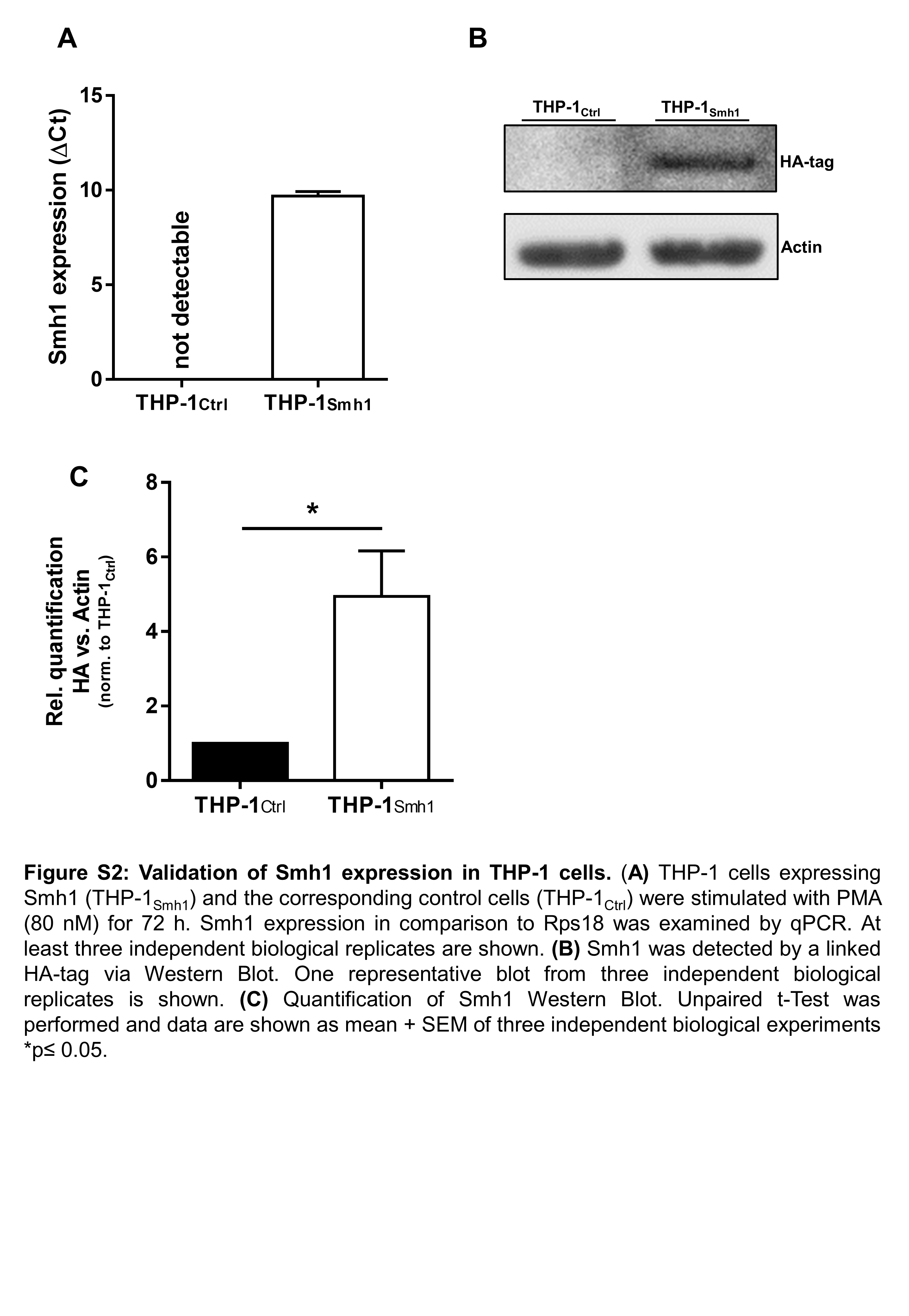

Supplement: Supplemental Material [file KVIR_A_2149973_SM9859.zip › supplementary/FigureS2.TIF]

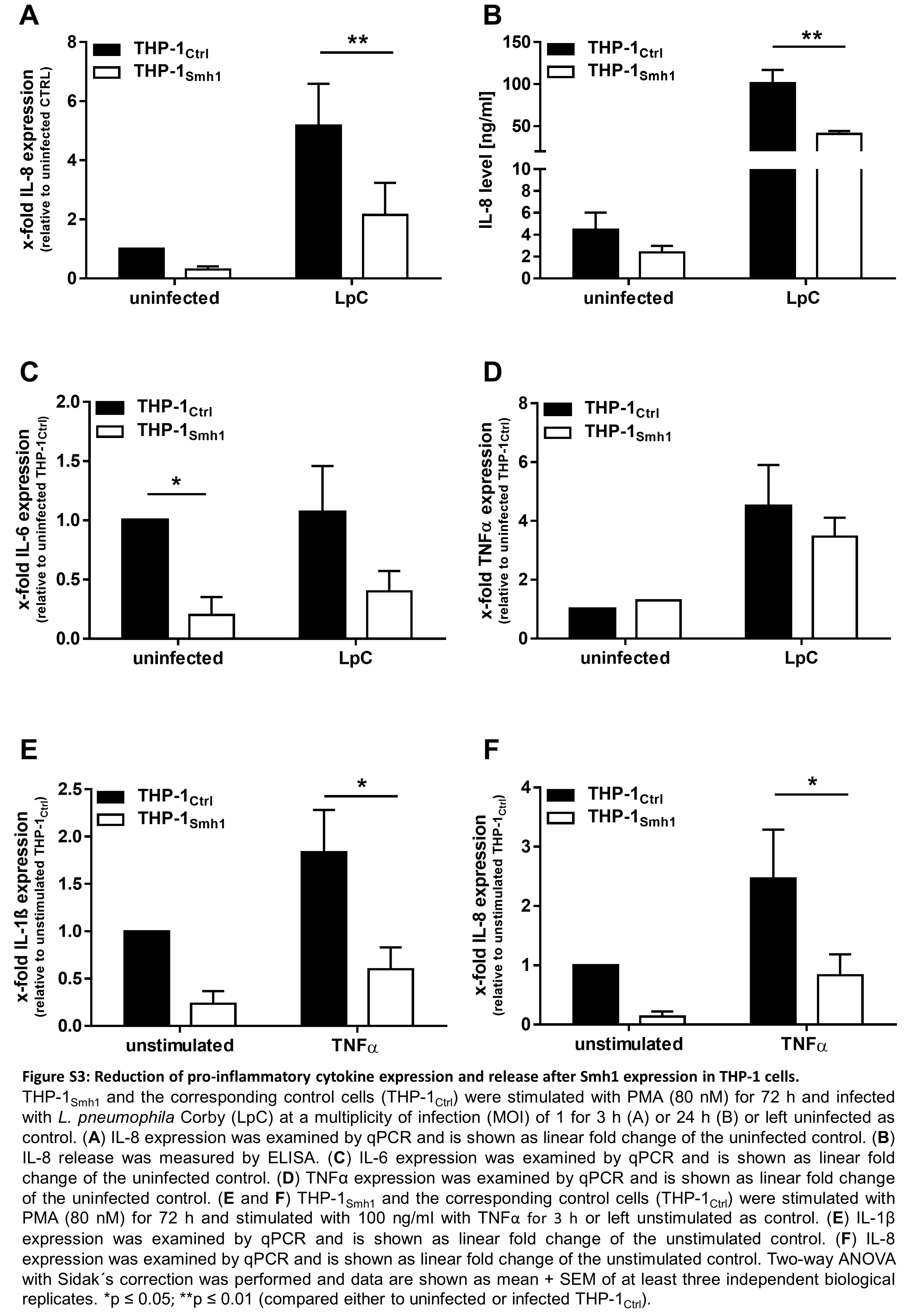

Supplement: Supplemental Material [file KVIR_A_2149973_SM9859.zip › supplementary/FigureS3.TIF]

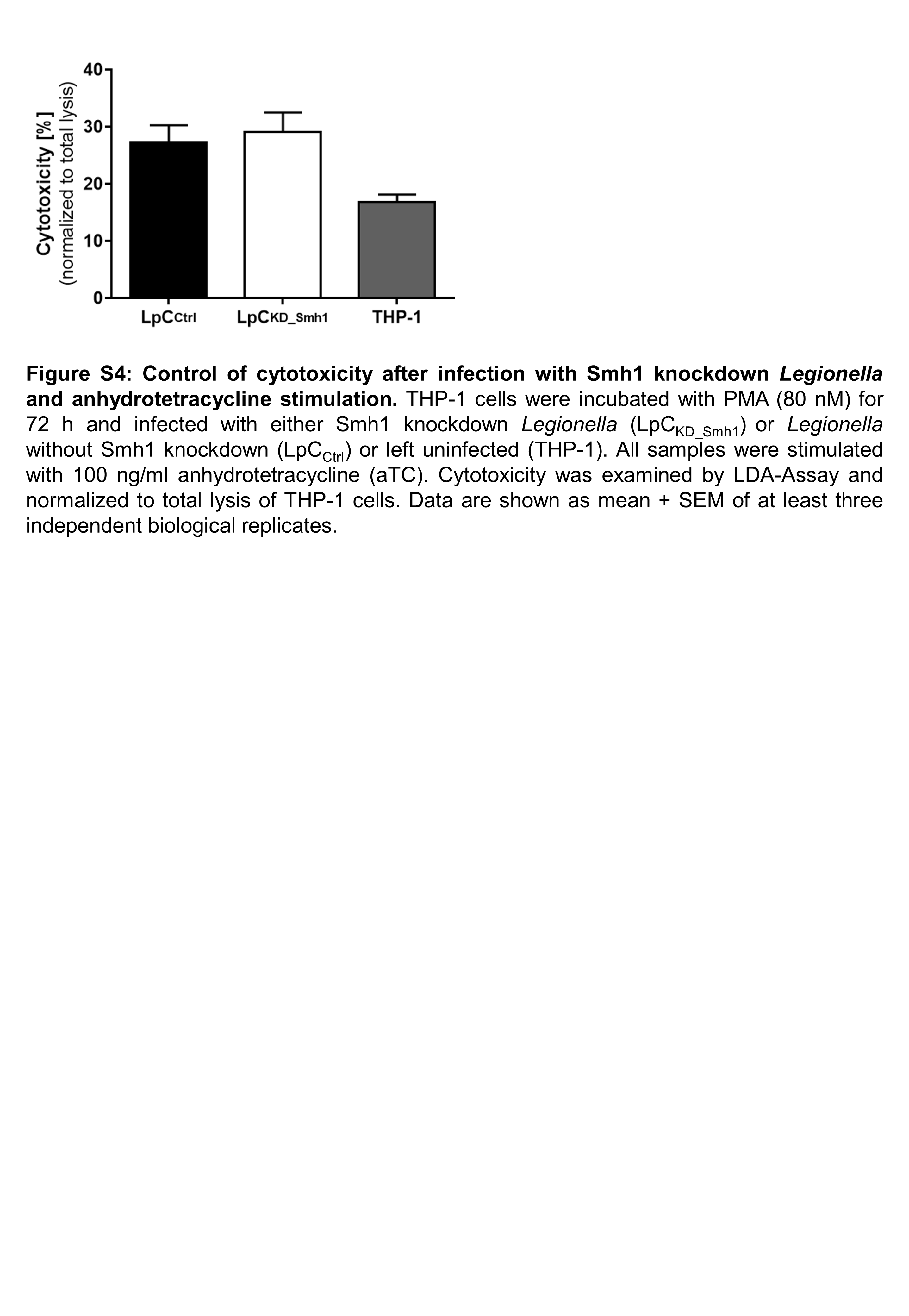

Supplement: Supplemental Material [file KVIR_A_2149973_SM9859.zip › supplementary/FigureS4.TIF]

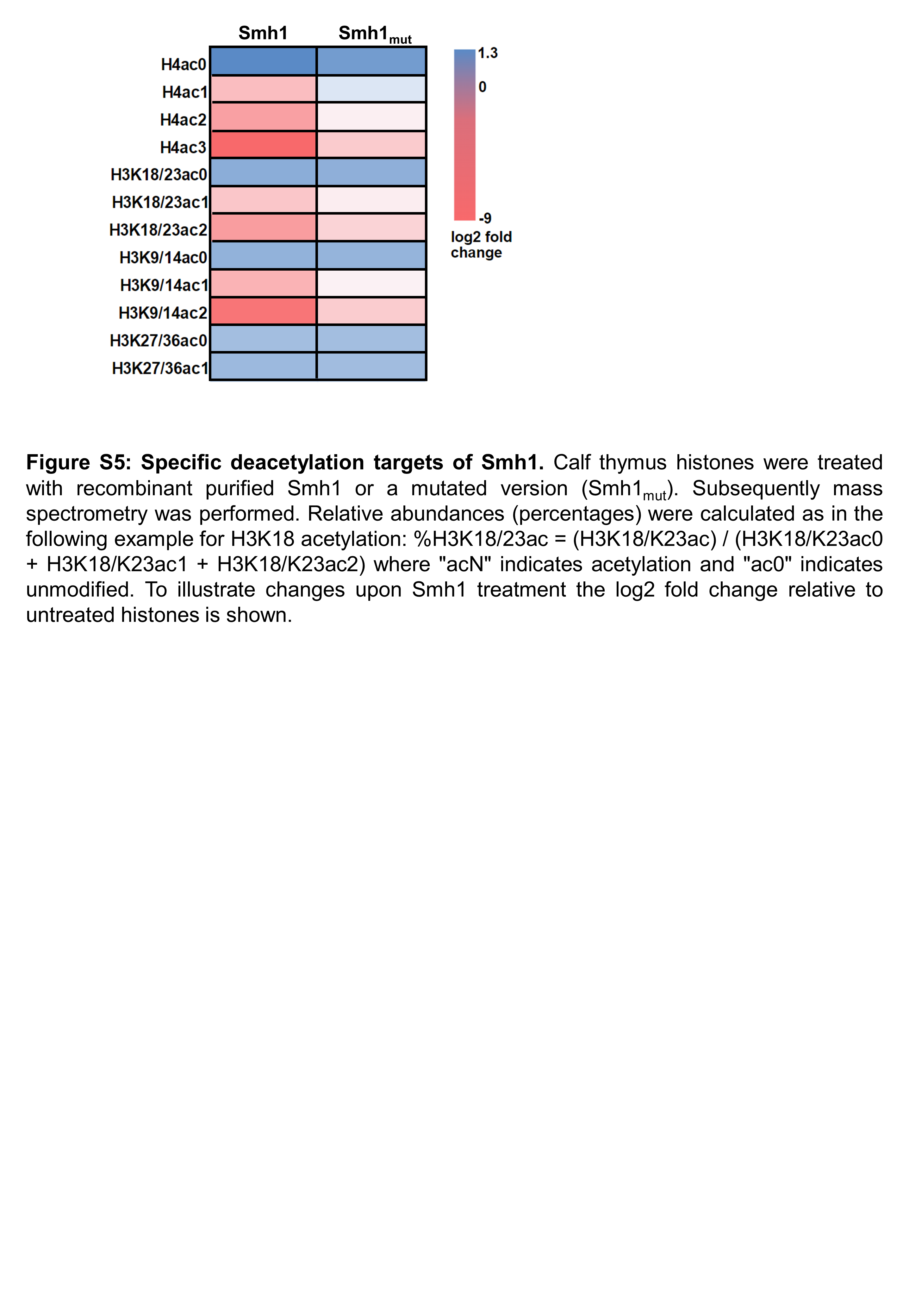

Supplement: Supplemental Material [file KVIR_A_2149973_SM9859.zip › supplementary/FigureS5.TIF]
